# Injury Mechanism, Volume, and Severity of General Surgical Trauma Patients During COVID-19 Lockdown

**DOI:** 10.7759/cureus.15914

**Published:** 2021-06-25

**Authors:** Ahmet Burak Çiftci

**Affiliations:** 1 General Surgery, University of Samsun, Samsun Training and Research Hospital, Samsun, TUR

**Keywords:** injury severity, covid-19 lockdown, activity restrictions, traumatic injury, general trauma surgery

## Abstract

Background

After the declaration of the COVID-19 pandemic, countries have taken many restriction measures to reduce the spread of the virus and ensure the health system's proper functioning. Our knowledge about the general surgery trauma patients being affected by the restrictions is very limited.

Objective

To examine the association of the lockdown measurements during the COVID-19 pandemic with general surgical trauma patients' volume and severity at a university teaching hospital.

Methods

All patients admitted to the emergency department because of trauma and evaluated by the general surgery team were examined in two groups. The COVID-19 restrictions period (17 March 2020 - 31 May 2020) and the corresponding time last year (17 March 2019 - 31 May 2019). Demographic properties, injury mechanisms, emergency trauma scores (ETS), hospital length of stays (HLOS), intensive care unit (ICU) admission rates, surgical interventions, and mortality were compared.

Results

The number of patients in the restrictions period is 30% lower than the before COVID-19 cohort. ETS was significantly higher in the restrictions period compared to the previous year, whereas no significant difference was detected in terms of injury mechanisms between the groups (p=0.001 vs p=0.493, respectively). HLOS was found to be higher in the restrictions period (p=0.038).

Conclusions

Although there was a decrease in the number of general surgical trauma admissions, a significant increase in the severity of trauma was observed during COVID-19 restrictions. We hope these findings will help authorities to guide resource allocation in future pandemic waves.

## Introduction

The coronavirus disease 2019 (COVID-19) disease caused by severe acute respiratory syndrome coronavirus 2 (SARS-CoV-2) is a pandemic that has affected most people in the world and caused more deaths than the influenza pandemic in 1918 [1]. According to official figures, over three million people have died because of COVID-19 and tens of millions of people have been infected [2]. In the early stages of the epidemic and later in fluctuation periods, many countries around the world have implemented intense restrictions and quarantine rules to control the disease and keep the health system in proper working order. Along with the restrictions, recommendations for staying at home and the concepts of social distance are frequently emphasized. Because of these restrictions and patients' fear of confronting COVID-19, dramatic reductions have been recorded in admissions to emergency departments and hospitals [3-5].

One of the leading reasons for emergency admissions is traumatic injuries. In the literature, many studies reported that there was also a decrease in admissions to the emergency departments attributable to trauma during the COVID-19 pandemic [6-9]. The vast majority of these studies were associated with orthopedic injuries and did not contain abdominal trauma patients. Also, studies examining isolated abdominal injuries and multisystem trauma cases were not encountered in the literature.

On 11 March 2020, when the first coronavirus patient was officially announced in our country, Turkey, restriction measures were immediately taken, and these restrictions were gradually narrowed after the declaration of the first COVID-related death on 17 March 2020 [10]. This study aimed to investigate how general surgery trauma patients were affected by restrictions during the COVID-19 lockdown period. The primary outcome of this study was to evaluate the volume, severity, and injury patterns of general surgical trauma patients during the COVID-19 lockdown and compare them with the corresponding time last year.

## Materials and methods

This study was conducted at the University of Samsun, Samsun Training and Research Hospital, which serves as a tertiary medical center in its region. The hospital has a 450-bed capacity and is regarded as a referral center for high-risk trauma patients. As it was declared a pandemic hospital, by the end of March, all elective and non-urgent surgical operations, except cancers, were postponed. During the pandemic, more than half of our hospital's bed capacity, including intensive care unit beds, was reserved for COVID-19 patients.

In this retrospective single-center study, we reviewed general surgical trauma patients admitted to our emergency department using hospital electronic database records. All trauma patients over 18 years of age with isolated abdominal or multisystem injuries were included in this study. Patients with isolated orthopedic injuries or isolated head/neck injuries were excluded. We analyzed patients in two different periods; the COVID-19 restrictions period (2020, lockdown period, 'after COVID-19' cohort) and the corresponding time last year (2019, 'before COVID-19' cohort). The COVID-19 restrictions period was between 17 March 2020 and 31 May 2020. Besides, the before COVID-19 period was determined as the same interval in the previous year. The reason why March 17 was chosen as the starting date was that strict social restrictions and quarantine rules started to be applied in our country from this date. 17 March was the date that strict social restrictions and quarantine rules were applied and 31 May was the date that the restrictions began to be relaxed and the so-called new normal period started in our country.

﻿We collected data including demographic characteristics such as age and sex, injury data (mechanism of trauma, emergency trauma score [ETS], and triage codes), and patient outcomes (hospitalization rates, intensive care unit requirements, emergency surgical interventions, hospital length of stay [HLOS], and in-hospital-mortality). Age was categorized as age groups <65 years and >65 years of age because patients over the age of 65 were the group most exposed to restrictions during this closure period. Mechanisms of injury were categorized as penetrating (gunshot wound or stab wound), assaults, traffic accidents (inside/outside the vehicle), and falls (from height or standing). Emergency surgical interventions were categorized under five main headings: small bowel injury (resection/primary repair), large bowel injury (resection/primary repair), splenic injury (splenectomy), suspected abdominal trauma (exploratory laparotomy), and others. Others included distal pancreatectomy, portal venous injury-lateral venorrhaphy, control of mesenteric bleeding, and suturing the liver lacerations.

In this study, the severity of the patients was determined by two parameters - ETS and the triage code at admission. ETS is accepted as a useful and easy tool for predicting mortality [11]. Due to its wide acceptance around the world and rapidly obtained results, we preferred to use it in this work. On the other hand, in our hospital, a triage system is applied in line with the Ministry of Health guidelines. In this system with four sub-categories, the red group has a high priority and indicates a life-threatening situation [5]. The yellow color indicates patients who have the potential for seriousness and need intervention within one hour at the latest. The green category refers to minor conditions that present as an outpatient and can be treated on an outpatient basis. The black color is the triage category used in case of death. So, we collected the triage categories of the patients to determine the severity of trauma and compared the red categories with yellow and green ones.

﻿This study was reviewed by the Non-Invasive Clinical Investigations Ethics Committee of the University of Health Sciences, Samsun Training, and Research Hospital, and received approval (protocol number: GOKA/2021/9/1).

Statistical analyses

Statistical analysis was performed using SPSS Statistics version 23 (IBM Corp., Armonk, USA). Data were tested for normality using the Shapiro-Wilk test. Categorical variables were defined as the number/total (%) and other descriptive data were presented as median [interquartile range (IQR)] (25%-75%). Chi-squared or Fisher's exact test was used for nominal outcomes. Mann-Whitney U test was used for continuous, non-normal data. A p-value of <0.05 regarded as the level of statistical significance.

## Results

A total of 124 general surgical trauma patients were identified across the two periods: 73 in the year 2019 cohort and 51 in the 2020 lockdown cohort. The number of patients in the lockdown period was 30% lower than the before-COVID-19 cohort. The median age at admission was 50 years (IQR 37-64) in the year 2019 and 45 years (IQR 32-58) in 2020, lockdown. There was no significant difference between the groups in terms of age and sex (p>0.05). While the rate of patients over 65 years old was 23.3% in the 2019 group; this rate decreased to 19.6% in the 2020 lockdown group. However, this reduction did not reach a statistical significance (p>0.05). Demographic data of general surgical trauma patients are presented in Table 1.

**Table 1:** Demographic data of general surgical trauma patients Values are numbers and (percentages) unless otherwise specified. *Values are median and (interquartile ranges); ^a^Mann-Whitney U test; ^b^Pearson Chi-square test.

	Before COVID-19 (n=73)	After COVID-19 (n=51)	p-value
*Age	50 (37-64)	45 (32-58)	^a^0.188
Age groups			^b^0.625
<65y	56 (76.7%)	41 (80.4%)	
>65y	17 (23.3%)	10 (19.6%)	
Sex			^b^0.567
Male	45 (61.6%)	34 (66.7%)	
Female	28 (38.4%)	17 (33.3%)	

The rate of patients who applied with the red triage code, which is a high-priority indicator, doubled in the 2020 lockdown group compared to the previous year (17.6% vs 8.2%). But no significant difference was detected between the groups in terms of overall triage colors (p>0.05). The median emergency trauma score was 1 (IQR 0-2) in the year 2019 group, whereas in the lockdown group it was 2 (IQR 1-4) (p=0.001).

When evaluating the mechanisms of injury, there was no significant difference between the groups (p>0.05). Traffic accidents were the leading mechanism of trauma during both periods. All trauma mechanisms were reduced during the lockdown period except for assaults. The most marked reduction was seen in the stab wounds and gunshot wounds group (53% reduction in lockdown period).

Hospitalization rates, the number of intensive care unit admissions, and emergency surgical interventions were similar between the groups (p=0.593, p=0.631, and p=0.757, respectively). In-hospital mortality rates were also identical before and after COVID-19 lockdown (5.5% vs 5.9% and p=0.609). Whereas the length of hospital stay was significantly higher in the lockdown group (p<0.05). Volume and characteristics of general surgical trauma patients before and after the COVID-19 periods are depicted in Table 2.

**Table 2:** Volume and characteristics of general surgical trauma patients before and after COVID-19 periods Values are numbers and (percentages) unless otherwise specified. *Values are median and (interquartile ranges); **Values show significance​​​​; ^a^Mann-Whitney U test; ^b^Pearson Chi-square test; ^c^Fischer’s exact test. ICU: intensive care unit.

	Before COVID-19 (n=73)	After COVID-19 (n=51)	p-value
Triage			^b^0.113
Yellow and Green	67 (91.8%)	42 (82.4%)	
Red	6 (8.2%)	9 (17.6%)	
*Emergency Trauma Score	1 (0-2)	2 (1-4)	^a^0.001**
Mechanism of injury			^b^0.493
Traffic accident	29 (39.7%)	24 (47.1%)	
Fall	25 (34.2%)	14 (27.5%)	
Stab wound+ Gunshot wound	13 (17.8%)	6 (11.8%)	
Assault	6 (8.2%)	7 (13.7%)	
Hospitalization	45 (61.6%)	29 (56.9%)	^b^0.593
*Length of hospital stay (days)	2 (0-4)	5 (0-7)	^a^0.038**
ICU admission	20 (27.4%)	16 (31.4%)	^b^0.631
Surgical intervention	13 (17.8%)	8 (15.7%)	^b^0.757
In-hospital-mortality	4 (5.5%)	3 (5.9%)	^c^0.609

While interventions for small bowel injuries were the leading cause in the 2019 group, splenectomies due to splenic injuries were the most common surgical intervention in the 2020 lockdown group (46.6%, 30% respectively). The comparison of emergency surgical interventions by period is shown in Table 3.

**Table 3:** Comparison of emergency surgical interventions by periods ^a^Other included distal pancreatectomy, portal venous injury-lateral venorrhaphy, control of mesenteric bleeding, and suturing the liver wounds. The reason for the difference in the total number of operations from the numbers in the other table is the intervention for more than one anatomical region in one patient.

	Before COVID-19	After COVID-19
Small bowel injury (resection/primary repair), n (%)	7 (46.6%)	2 (20%)
Large bowel injury (resection/primary repair), n (%)	2 (13.3%)	2 (20%)
Splenic injury (splenectomy), n (%)	2 (13.3%)	3 (30%)
Suspected abdominal trauma (exploratory laparotomy), n (%)	2 (13.3%)	1 (10%)
^a^Other, n (%)	2 (13.3%)	2 (20%)
Total	15	10

## Discussion

With this study, we examined the association of the lockdown measures during the COVID-19 pandemic with general surgical trauma patients' volume and severity at a university-affiliated teaching hospital. We found a dramatic reduction in the number of general surgical trauma patients in the lockdown period compared to the previous year, whereas the severity of diseases was significantly increased during the lockdown.

The overall decrease in traumatic admissions suggests that people follow quarantine rules, lockdown principles, and stay-at-home recommendations during the first wave of the pandemic. On the other hand, ETS was significantly increased in the lockdown period. This finding can be explained by the fact that some mildly severe cases prefer not to apply to the hospital due to fear of COVID-19 transmission and to recover on their own. Also, we think that these patients did not visit in the early post-traumatic period and may have applied to the hospital when their condition worsened, and therefore they had higher trauma scores. In this study, the number of patients in the red triage category was higher in the lockdown group (8.2% vs 17.6%, before covid and after covid). It can be said that this situation also leads to an increase in the emergency trauma score. Therefore, being in the red category has been associated with an increase in trauma severity. Considering the studies examining the severity of trauma patients during the lockdown period, it was seen that the injury severity score (ISS) was used in these articles and it was emphasized that the ISS scores did not change with the pandemic lockdown period [12-14]. Unlike these studies, the increased severity of trauma patients in our study was considered as the absence of isolated orthopedic and isolated head trauma patients in the patient group included in this work.

We found no significant difference between groups in terms of mechanisms of injury. This finding was consistent with other studies in the literature [12-14]. But Yeates et al. found an increase in gunshot wounds after COVID-19 stay-at-home advisories [15]. In another study conducted on this subject, it was emphasized that traumas due to falls increased and other trauma mechanisms decreased during the COVID-19 pandemic [16].

In our study, the HLOS was significantly higher in the lockdown group. The higher trauma scores of the patients at their admissions to the emergency are considered as a reason for this situation. In addition, possible complications related to COVID-19 disease may have prolonged this time. Different results have been found in studies in the literature on this subject. In contrary to our results, Yeates et al. found a shorter length of stay in the post-stay-at-home order group [15]. Ghafil and colleagues reported no differences in mean HLOS between the groups [7]. And Devarakonda et al., like our findings, stated that mean HLOS increased in the COVID-19 period [14].

When considering mortality rates, we found no significant difference between the groups. This finding was consistent with other studies in the literature [7,12,15,16]. Also, ICU admissions and surgical intervention rates were similar in this study.

When emergency surgical interventions due to trauma were examined, repairs or resections of small bowel injuries were the most commonly encountered operations in the before-COVID-19 group. Whereas, in the after-COVID-19 group, splenectomies took first place.

To the best of our knowledge, this was the first study evaluating the general surgical trauma patients' volume and severity during the COVID-19 lockdown. Also, there was no published data evaluating the surgical intervention types of general surgical trauma patients during the lockdown. These features were the strength of our study. On the other hand, there were some limitations of this study. First, a small sample size may affect the results of the study. Second, this study was retrospective in nature, so some patient data may have been ignored or may not be available during the study period. Lastly, this was a single-center study and may not be applicable to different parts of the country.

## Conclusions

This study's findings illustrate the significant diminishing effect of the COVID-19 lockdown measures on general surgery trauma patients' admissions to the emergency department. Although the total admissions due to trauma decreased, more serious trauma cases were encountered by the general surgery team during the COVID-19 closure period. We hope these findings will help authorities to guide resource allocation in future waves of new pandemics.
